# Methicillin-Resistant Staphylococcus Aureus Bacteremia: Epidemiology, Clinical Characteristics, Risk Factors, and Outcomes in a Tertiary Care Center in Riyadh, Saudi Arabia

**DOI:** 10.7759/cureus.14934

**Published:** 2021-05-10

**Authors:** Sarah A Alhunaif, Sarah Almansour, Raghad Almutairi, Sara Alshammari, Lamia Alkhonain, Bassam Alalwan, Sameera Aljohani, Abdulrahman Alsaedy, Mohammad Bosaeed, Adel Alothman

**Affiliations:** 1 College of Medicine, King Saud bin Abdulaziz University for Health Sciences, Riyadh, SAU; 2 Division of Infectious Diseases, Department of Internal Medicine, King Abdulaziz Medical City - National Guards Health Affairs, Riyadh, SAU; 3 Department of Pathology & Laboratory Medicine, King Abdulaziz Medical City - National Guards Health Affairs, Riyadh, SAU; 4 Department of Infectious Diseases, King Abdullah International Medical Research Center, Riyadh, SAU

**Keywords:** methicillin resistant staphylococcus aureus (mrsa), incidence, risk factors, mortality, bacteremia, methicillin resistant staphylococcus aureus bacteremia

## Abstract

Background

Methicillin-resistant *Staphylococcus aureus* (MRSA) has become increasingly common in hospitals worldwide. In an era of pandemics, it is important to understand all types of infectious diseases in order to control its outcome and reduce mortality rates related to it. This study aims to identify the epidemiology of MRSA bloodstream infection, risk factors, and mortality in King Abdulaziz Medical City in Riyadh.

Methods

This is a retrospective chart review study from January 2013 to June 2017. All patients with positive blood culture for MRSA at King Abdulaziz Medical City (KAMC), which is a tertiary care center in Riyadh, Saudi Arabia, were included. Data were extracted from electronic and paper medical records for patients who had a positive blood culture for MRSA. Data collected included demographics, microbiological details, risk factors, and 30-day mortality.

Results

From 2013 through June 2017, 633 *Staphylococcus aureus *bacteremia were reported in KAMC, of which 184 (29.1%) were MRSA with a mean age of 60±19 years. Almost all of our isolates were susceptible to vancomycin, linezolid, and tigecycline. The most common infectious syndrome these patients presented with was an endovascular infection which accounts for 30.4% while 19.9% presented as a case of pneumonia. The mortality within 30 days of collection of the positive blood culture was 20.65%. Male gender (OR = 2.33; 95% CI = 1.34-4.05; P-value = 0.003) and patients with history of recent hospital (OR = 2.34; 95% CI = 1.27-4.34; P-value = 0.007) or ICU (OR = 1.66; 95% CI = 1.09-2.52; P-value = 0.018) admissions were more likely to acquire MRSA.

Conclusions

The incidence of MRSA bacteremia at King Abdulaziz Medical City is high and increasing in conjunction with incidence rate posing a significantly high rate of mortality. Recent history of hospital admission, ICU admission and males were found to be significantly associated with higher rates of MRSA infection. Some modifiable risk factors that could be used to facilitate the reduction of MRSA acquisition rates is to avoid unnecessary hospital and ICU admissions.

## Introduction

Methicillin-resistant *Staphylococcus aureus* (MRSA) is a significant global healthcare problem associated with remarkable morbidity and mortality among infected patients. MRSA accounts for most of the global *Staphylococcus aureus* (*S. aureus*) bacteremia cases. In comparison with methicillin-sensitive *S. aureus *(MSSA), MRSA infections have a higher rate of undesirable clinical outcomes including prolonged hospital stay, metastatic infections such as infective endocarditis, septic arthritis, osteomyelitis, and death [1]. An Australian study was conducted for over one year concluded that 450 of 1994 cases (24%) of S. aureus bacteremia were due to MRSA. Most importantly, this study showed that 30-day mortality was 30% due to MRSA compared with 17.7% for MSSA [2].

MRSA bacteremia has majorly increased in recent years due to the increased prevalence of invasive procedures, increased numbers of immunosuppressed patients, as well as the increased resistance to antibiotics [3]. Many studies have found that the prevalence of MRSA is influenced by geographical location. It is now as high as up to 60% in certain centers in the United States [4], and more than 70% in a report from Shanghai, [5] but great geographic variations exist worldwide. In European centers, these percentages varied from less than 2% in the Netherlands to 54.4% in Portugal [6]. In the US hospitals, 60% of *S. aureus* isolates are found in the intensive care unit (ICU) [7]. MRSA in non-ICU patients annual rate in the USA has increased during the period 1998-2005 to 59.2% [4]. Several studies showed several factors associated with *S. aureus* bacteremia including previous MRSA infection or colonization, skin ulcers or cellulitis at hospital admission, the presence of a central venous catheter, urinary catheter insertion, skin and soft tissue infection, intravenous drug abuse, the presence of the immunocompromising condition, use of corticosteroid and liver disease [8].

In Saudi Arabia, a meta-analysis was done for epidemiological data from 26 studies from across Saudi Arabia during 2002-2012. It showed that MRSA prevalence rate increased from 2% in 1988 reaching up to 38% in 2012 [9]. Meanwhile, an older study was conducted in 1998 at King Abdulaziz University Hospital in Jeddah, Saudi Arabia reported that 74.8% of MRSA isolates were obtained from hospitals. Surgical wounds (31.1%), the chest (27%), and endovascular catheters (20.3%) are frequently the common sites of infection [10].

This study aims to determine the epidemiology of MRSA bacteremia from 2013 following up to 2017, by describing the demography and clinical characteristics of patients, identifying modifiable risk factors, and estimating the 30-day mortality.

## Materials and methods

Design and data collection

This is a retrospective cohort study consisting of 184 blood samples positive for MRSA in King Abdulaziz Medical City in Riyadh, which is a tertiary care center with a bed capacity of approximately 1,200 beds serving a steady population of the national guard, military personnel, and civilians and their dependents. All adult patients with positive blood culture for MRSA who were admitted were included in the study. We collected all samples between January 2013 until June 2017. Patient characteristics included gender, age, patient’s comorbidities, recent hospital admission, Intensive care unit admissions, length of hospitalization, department of admission, an infectious syndrome that the patient was diagnosed with and the presence of a prosthetic device or central venous catheter, microbiological data and mortality. All data collected was archived by the institutional review board with access provided on request. An episode of MRSA bacteremia was defined as any blood culture positive with MRSA. Episodes occurring within two weeks of a previous one, or without resolution of signs and symptoms of infection after a previous episode, were considered as single episodes. An intravascular device (IVD) was thought the likely focus of infection if there was evidence of inflammation at the catheter insertion site and/or catheter tip culture was positive for MRSA by a semi-quantitative method. Endocarditis was defined according to the modified Duke’s criteria. Pneumonia was defined as the source of bacteremia in the presence of the purulent sputum from which MRSA was isolated and new lung field opacity on radiographic examination.

Statistical analysis

All data were analyzed using R software version 4.0.2 (R Foundation for Statistical Computing, Vienna, Austria) [11] using the packages (Rcmdr) [12] and (glm2) [13]. Categorical variables were represented as frequencies, and percentages with Chi-square test (or Fisher’s exact test, as appropriate) was used for testing the gender differences. For continuous variables, the representation was as means and standard deviations, using skewness and Kurtosis tests to evaluate the normal distribution of the variables. Based on normality status, independent-samples t-test or Mann-Whitney U-test were used to compare females to males. Moreover, we used univariate logistic regression to identify any possible modifiable risk factors for MRSA. Logistic regression results were expressed as odds ratios (ORs) and 95% confidence interval (95% CI). A P-value of ≤ 0.05 was considered statistically significant in all analyses.

## Results

During the study period, a total number of 633 *S. aureus* isolates were isolated from blood; of which 184 (29.1%) were MRSA. Among MRSA patients, the number of male patients was 124 (67.4%) whereas female patients 60 (32.6%), with a mean age of 60±19 years. Regarding co-morbidities, 127 (69.0%) of the patients were hypertensive, 120 (65.2%) were diabetics, 57 (31.0%) were suffering from chronic kidney disease, 26 (14.1%) were diagnosed with cancer, 8 (4.3%) were on long-term corticosteroid usage and 4 (2.2%) had an autoimmune disease. It should be noted that 45.7% of the patients have a history of recent hospital admissions during the last 90 days and 66 (35.9%) patients had documented a previous history of MRSA colonization and/or infection. (Table 1).

**Table 1:** Summary of baseline characteristics of the included patients (N = 184). MRSA: methicillin-resistant *Staphylococcus aureus*; N: numbers; SD: standard deviation. *Statistically significant.

Variables		Previous history of colonization/infection with MRSA				Total		P-value
		Yes		No				
		n	%	n	%	n	%	
Age; mean ± SD		57 ± 18		61 ± 19		60 ± 19		0.135
Gender	Male	48	72.7	76	64.4	124	67.4	0.248
	Female	18	27.3	42	35.6	60	32.6	
Year of admission	2013	11	16.7	24	20.3	35	19.0	0.507
	2014	12	18.2	19	16.1	31	16.8	
	2015	4	6.1	16	13.6	20	10.9	
	2016	26	39.4	38	32.2	64	34.8	
	2017	13	19.7	21	17.8	34	18.5	
Diabetes mellitus	Yes	43	65.2	77	65.3	120	65.2	0.989
	No	23	34.8	41	34.7	64	34.8	
Hypertension	Yes	49	74.2	78	66.1	127	69.0	0.252
	No	17	25.8	40	33.9	57	31.0	
Chronic kidney disease	Yes	21	31.8	36	30.5	57	31.0	0.854
	No	45	68.2	82	69.5	127	69.0	
On long-term corticosteroids	Yes	4	6.1	4	3.4	8	4.3	0.460
	No	62	93.9	114	96.6	176	95.7	
Autoimmune disease	Yes	1	1.5	3	2.5	4	2.2	1.00
	No	65	98.5	115	97.5	180	97.8	
Cancer	Yes	10	15.2	16	13.6	26	14.1	0.766
	No	56	84.8	102	86.4	158	85.9	
History of recent hospital admission (last 90 days)	Yes	39	59.1	45	38.1	84	45.7	0.006*
	No	27	40.9	73	61.9	100	54.3	

The type of infectious syndrome varied among the population study, 56 (30.4%) were presented as cases of endovascular infection such as septic thrombophlebitis, endocarditis, central venous catheter infections. Pneumonia was reported in 36 (19.6%) of the patients, 35 (19.0%) of the patients were presented with skin and soft tissue infections, 24 (13.0%) had bone and/or joint infection, 19 (10.3%) with urinary tract infection, and in 12 (6.5%) patients there was no clear source of infection identified. At the time of diagnosis, 88 (47.8%) were having a central venous catheter, 11 (6.0%) had implanted a medical prosthetic device, and 52 (28.3%) had surgery during the same admission (Table 2).

**Table 2:** Summary of clinical characteristics of the patients (N = 184). UTI: urinary tract infection; CNS: central nervous system; N: numbers; *including: cardiac device, prosthetic valve, knee prosthetic joint, vertebrae prosthesis; SD: standard deviation.

Variable		N	%
Specialty	Medicine	132	71.7
	Surgery	35	19.0
	Obstetrics	1	0.5
	Oncology	16	8.7
Infectious syndromes	Pneumonia	36	19.6
	Endovascular	56	30.4
	Bone/joint	24	13.0
	Skin and soft tissue	35	19.0
	UTI	19	10.3
	CNS	2	1.1
	Unknown source	12	6.5
Central venous catheter	Yes	88	47.8
	No	96	52.2
Implanted medical prosthetic device*	Yes	11	6.0
	No	173	94.0
Had surgery during the same admission	Yes	52	28.3
	No	132	71.7
Length of hospitalization (days); mean ± SD		55 ± 112	

**Figure 1:**
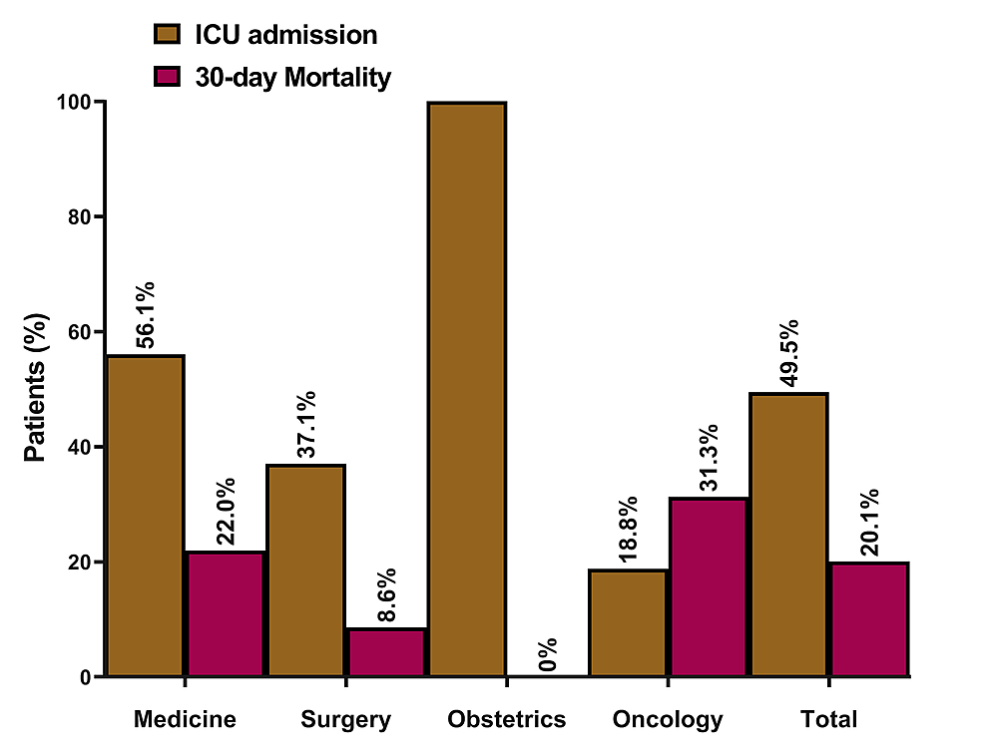
MRSA patients’ outcomes (ICU admission and 30-day mortality), stratified by admitting specialties (N = 184). MRSA: methicillin-resistant *Staphylococcus aureus;* ICU: intensive care unit; N: numbers.

A total of 91 (49.46%) of the patients were admitted to the intensive care unit at the time of diagnosis. The majority of the patients were admitted under medical specialty 132 (71.74%), 36 (19.56%) under surgical specialty, 16 (8.7%) under hematology/oncology service and 1 patient admitted with obstetrics and gynecology (0.5%). The 30-day mortality for the patients with MRSA bacteremia was (20.1%) 35/174, 10 patients were lost to follow-up (Figure 1).

**Figure 2:**
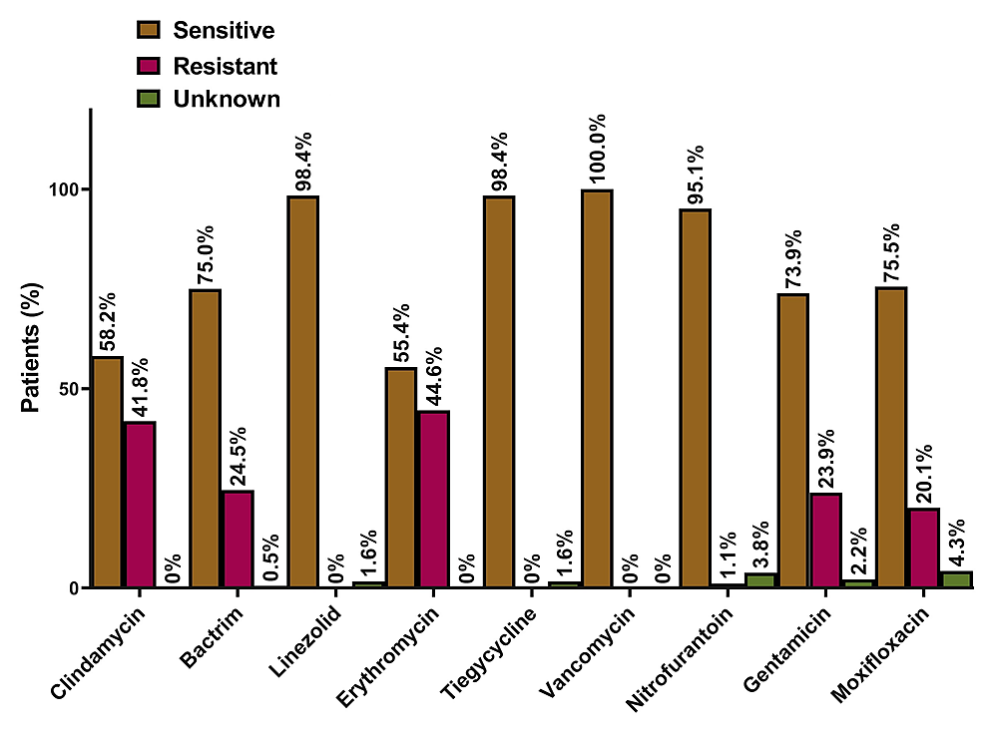
Antimicrobial susceptibility of MRSA isolates (N = 184). MRSA: methicillin-resistant *Staphylococcus aureus*; N: numbers.

Almost all MRSA isolates in our study were susceptible in vitro to vancomycin, linezolid, and tigecycline. Susceptibility to other antibiotics was variable with 95.1% of the isolates susceptible to nitrofurantoin, 75.5% to moxifloxacin, 73.9% to gentamicin, 58.2% to clindamycin, and 55.4% to erythromycin (Figure 2).

Table 3 presents results from the univariate logistic analysis of different factors among MRSA and non-acquisition groups. On one hand, male gender (OR = 2.33; 95% CI = 1.34-4.05; P-value = 0.003) and patients with history of recent hospital (OR = 2.34; 95% CI = 1.27-4.34; P-value = 0.007) or ICU (OR = 1.66; 95% CI = 1.09-2.52; P-value = 0.018) admissions were more likely to acquire MRSA. On the other hand, patients who were on long-term corticosteroids (OR = 0.54; 95% CI = 0.40-0.74; P-value < 0.001), those with an autoimmune disease (OR = 0.57; 95% CI = 0.42-0.77; P-value < 0.001), and those who had surgery during the same admission (OR = 0.31; 95% CI = 0.16-0.60; P-value < 0.001) showed lower frequency of MRSA infections. In contrast, age, co-morbidities, cancer, admitting specialty, having central venous catheter, or implanted medical prosthesis did not show any significant association with MRSA rates.

**Table 3:** Univariate logistic regression of potential risk factors for methicillin-resistant Staphylococcus aureus. SE: standard error; ICU: intensive care unit. *Statistically significant.

Predictor	Estimate	P-value	Odds ratio	95% confidence interval	
				Lower	Upper
Gender (male versus female)	0.85	0.003*	2.33	1.34	4.05
Age	-0.01	0.257	0.99	0.98	1.01
Diabetes mellitus	0	0.989	1	0.53	1.87
Hypertension	0.39	0.253	1.48	0.76	2.89
Chronic kidney disease	0.06	0.854	1.06	0.56	2.04
On long-term corticosteroids	-0.61		0.54	0.40	0.74
Autoimmune disease	-0.57		0.57	0.42	0.77
Cancer	0.13	0.766	1.14	0.48	2.68
History of recent hospital admission (last 90 days)	0.85	0.007*	2.34	1.27	4.34
Admitting specialty	0.07	0.663	1.08	0.77	1.51
ICU admission	0.51	0.018*	1.66	1.09	2.52
Central venous catheter	-0.23	0.454	0.79	0.43	1.45
Implanted medical prosthetic device	-0.02	0.972	0.98	0.28	3.47
Had surgery during the same admission	-1.17		0.31	0.16	0.60

## Discussion

The current study is one of the few studies on MRSA epidemiology in a tertiary-care Saudi setting. We conducted a longstanding cohort of all adult patients with positive blood culture for MRSA who were admitted to King Abdulaziz Medical City between January 2013 until June 2017. The results of our study showed that a high MRSA incidence of 29.1%, which is consistent with the reported incidence in literature, ranging from 7% to 60% [14].

Approximately one-third (30.4%) of cases presented as an endovascular infection, which represented the most common site of infection among the bacteremia patients followed by pneumonia and skin and soft tissue infection (19.9%) (19.3%), respectively. The increased susceptibility of having an endovascular infection could be related to the poor general condition of patients in the present study, as most patients had comorbidities, other reason would be the presence of intravascular devices in most of these patients. These results were similar to what has been observed by other researchers. For instance, in the Veterans Administration Medical Centre, Buffalo, New York, the commonest foci of *Staphylococcus aureus* bacteremia (SAB) were intravascular catheter (33%), postoperative wounds (11%), skin infections (7%), pulmonary infections (7%) [15]. Moreover, sensitivities to a selection of anti-staphylococcal antibiotics were available from routine automated testing. All isolates were sensitive to vancomycin, linezolid, and tigecycline and variable susceptibility to other agents. Similar results were observed in other studies [16].

Although the mean age of patients presented with bacteremia was 60 years, MRSA affected all age groups, which was also evident with age being an insignificant predictor for MRSA risk. Many previous studies of MRSA patients and healthy controls came to the same conclusion that age by itself may not be a significant risk factor for MRSA [17]. Contradicting with our findings, some other studies concluded that advanced age, especially those who are older than 65 years, are at more risk to acquire MRSA [18-21]. This may be explained by the increased risk of hospital admission and hence the risk of MRSA acquisition among senior age groups, which is not a direct effect [22,23]. In the same context, our results showed an increased risk of acquiring MRSA among male patients, which has been also confirmed by previous studies [18,20]. In one of the biggest cohorts of MRSA acquisition in an acute tertiary-care hospital, they found male gender to be a significant predictor (P-value < 0.001) of MRSA bacteremia, compared to females [18]. Since the inappropriate hygienic habits can increase the risk of horizontal transmission of infections, which is consistent with MRSA strains, this higher incidence among males could be a reflection of their lower hygienic standards [24].

History of recent hospital admission and ICU admission were both significant predictors of higher risk of MRSA acquisition. Many previous studies showed that hospitalization is a major risk factor for MRSA acquisition which acts in a dose-response manner, based on the length of hospital stay [17,18,25]. In Singapore, a large case-control study of 1200 patients showed that hospitalization had a direct effect on MRSA acquisition and the length of stay accounted for the most of effects caused by age (100%), surgical operations (96%), and immunosuppressive states (67%) [25]. Akin to that, ICU-admitted patients were at higher risk of nosocomial MRSA infections, compared to those admitted at non-ICU departments [20,26]. This may be explained by the increased need for mechanical ventilation in ICU-admitted patients, which is a known risk factor for ventilator-related pneumonia and, in turn, MRSA acquisition [27]. Other identified risk factors for the high prevalence in the ICU setting include inappropriate numbers of well-trained nurses and bad hygiene measures [26]. In the same context, our results showed a reduction in MRSA acquisition rates among patients with auto-immune diseases and those who are on a long-term corticosteroid. Although corticosteroid was found to increase the risk of S. aureus acquisition in general and MRSA in specific [28], many other studies, like the current one, did not show any significant increase in MRSA acquisition rates among corticosteroid users [17].

The mortality rate within 30 days after the MRSA bacteremia episode during the study period was (21.05%). Notably, we found that 30-day mortality has increased over time from (19.3%) in 2013 to (38.2%) in 2017 despite advancement in antimicrobial treatment. Our results were similar to a study that was done in a university hospital, Barcelona, Spain, which showed that the mortality of patients with MRSA bacteremia remains high, close to 30% [29]. Also, a large study carried out across England during 2004-2005 showed that 30-day mortality due to MRSA bacteremia was (38%) [30].

The limitations of the present study include the retrospective study design and the sensitivity of MRSA detection which is mainly dependent on the sensitivity of the culture. Nevertheless, these possible biases would not be eliminated when adopting the case and control groups. As limited by study design, there is a possible residual confounding and accordingly, the causation could not be established. Regarding our study strength, it is one of the largest cohort studies to be conducted in Saudi Arabia on MRSA acquisition, with a long-standing period of five years and taking into count many variables. Using patients’ structured medical records would ensure data accuracy/consistency and minimize measurement mistakes. All collected data were not liable to recall bias or protocol variation since none of them was based on patient reporting.

## Conclusions

The prevalence of MRSA bacteremia at King Abdulaziz Medical City is high and increasing and carries a significant rate of mortality. Recent history of hospital admission, ICU admission and males were found to be significantly associated with higher rates of MRSA infection. Some modifiable risk factors that could be used to facilitate the reduction of MRSA acquisition rates is to avoid unnecessary hospital and ICU admissions. Lastly, more studies are needed to explore the role of unmodifiable risk factors such as male gender in MRSA infection.
